# Supplementary material for: Dietary Arginine Supplementation Improves Intestinal Mitochondrial Functions in Low-Birth-Weight Piglets but Not in Normal-Birth-Weight Piglets
Source: Antioxidants (Basel). 2021 Dec 15;10(12):1995. doi: 10.3390/antiox10121995 (PMC8698761; doi:10.3390/antiox10121995)
Supplement: Supplementary file 1 [file antioxidants-10-01995-s001.zip › antioxidants-1479646-supplementary.pdf]

Supplementary Data

Supplemental Table S1. Dietary composition and nutrient levels

Item	%
Ingredients	
Whole milk powder	58.00
Whey protein concentrate	26.00
Casein	2.00
Glucose	1.18
Coconut oil	10.00
Choline chloride	0.10
Vitamin mixture[1]	0.10
Mineral mixture[2]	0.10
L-Lys.Hcl (78%)	0.26
DL-Met (98.5%)	0.11
L-Thr (98%)	0.10
L-Ala (99%)	2.05
Total	100.00
Nutrient content[3]	
DM	96.91
DE (MJ/kg)	21.05
CP (%)	27.34
Fat (%)	25.72
Lactose (%)	35.94
Ca (%)	0.96
Available P (%)	0.59
Total-Lys (%)	2.17
Total-Met (%)	0.82
Total-Thr (%)	1.35
Total-Trp (%)	0.52
Total-Arg (%)	0.85

[1] Provided the following per kilogram of diet: 12 000 IU retinol, 4 000 IU cholecalciferol, 40 mg dl-α-tocopherol, 4 mg menadione, 4 mg thiamin, 10 mg riboflavin, 0.16 mg biotin, 6 mg pyridoxine, 40 μg cyanocobalamin, 40 mg niacin, 20 mg pantothenic acid, 2 mg folic acid.

[2] Provided the following per kilogram of diet: 100 mg Fe (as $FeSO_4 \cdot 7H_2O$); 10 mg Cu (as $CuSO_4 \cdot 5H_2O$); 5 mg Mn (as $MnSO_4 \cdot H_2O$); 100 mg Zn (as $ZnSO_4 \cdot 7H_2O$); 0.3 mg Se (as $Na_2SeO_3 \cdot 5H_2O$); 0.2 mg I (as KI).

[3] Calculated values.

Supplementary Data

Supplemental Table S2. Sequences and parameters of primers for the real-time qPCR

Genes	Nucleotide sequence	Anneal temperature/℃	Genbank ID
ZO1	CTGAGGGAATTGGGCAGGAA<br>TCACCAAAGGACTCAGCAGG	59.7	XM_005659811.1
Occludin	CAGGTGCACCCTCCAGATTG<br>GGACTTTCAAGAGGCCTGGAT	59.7	NM_001163647.2
Claudin1	GCCACAGCAAGGTATGGTAAC<br>AGTAGGGCACCTCCCAGAAG	59.7	FJ873109.1
PGC-1α	CCCGAAACAGTAGCAGAGACAAG<br>CTGGGGTCAGAGGAAGAGATAAAG	59.7	NM_213963
NRF1	GCCAGTGAGATGAAGAGAAACG<br>CTACAGCAGGGACCAAAGTTCAC	59.7	AK237171.1
TFAM	GGTCCATCACAGGTAAAGCTGAA<br>ATAAGATCGTTTCGCCCAACTTC	55.8	NM_001130211.1
Cytc	AGTTGGCCACCGCCTTATTT<br>CCAACAGAAACATTCCATCAGCC	55.8	NM_001129970
Cox Ⅰ	ATTATCCTGACGCATACACAGCA<br>GCAGATACTTCTCGTTTTGATGC	59.7	AJ950517.1
CoxⅣ	CCAAGTGGGACTACGACAAGAAC<br>CCTGCTCGTTTATTAGCACTGG	59.7	AK233334.1
Cox Ⅴ	ATCTGGAGGTGGTGTTCCTACTG<br>GTTGGTGATGGAGGGGACTAAA	59.7	AY786556.1
FIS1	AGTAGTGAGGATTGCGAGGC<br>TACTTGCTTCGCACCAGACA	59.7	XM_021086263.1
OPA1	TGCCTGACATTGTGTGGGAG<br>AACCAATTTGTGACCTGTGGTG	59.7	XM_021070065.1
MFN 1	GAAAGCACAAAGCACAGGGG<br>CTTTTCTCTGCTGCTGCCAC	59.7	XM_021068494.1
MFN2	CCTCTGTTCCAGTTGTGCCA<br>CATGCACAGCAACTCCTAGC	59.7	XM_021095364.1
Drp1	GCAGCCATTTCACAAACCTGT<br>TTTGAGGGGTAGGGGGATTTC	59.7	NM_017426.4
β-actin	CCTGCGGCATCCACGAAAC<br>TGTCGGCGATGCCTGGGTA	55.8	XM_003124280.3
